# A fundamental model for oxygen consumption of Atlantic salmon

**DOI:** 10.1038/s41598-026-47328-6

**Published:** 2026-04-13

**Authors:** André Morin, Terje Jacobsson, Tim Dempster, Fletcher Warren-Myers, Frode Oppedal, Malthe Hvas

**Affiliations:** 1 Sustainable Aquaculture Laboratory – Temperate and Tropical (SALTT), Deakin University, Geelong, Australia; 2 Department of Engineering Cybernetics, Faculty of Information Technology and Electrical Engineering, Norwegian University of Science and Technology, Trondheim, Norway; 3 Animal Welfare Research Group, Institute of Marine Research, Matre, Norway

**Keywords:** Allometric, Bioenergetics, Metabolic Rate, Swim Tunnel Respirometry, Temperature, Ecology, Ecology, Ocean sciences

## Abstract

Predicting oxygen availability in Atlantic salmon farms is challenging, but digital simulations that couple bioenergetics and hydrodynamics show great promise. Robust simulations depend on reliable estimates of oxygen demand, yet previous empirical models offer limited accuracy. Here, we present a refined fundamental model for Atlantic salmon oxygen consumption rate (MO_2_) as a function of three readily measurable parameters: body weight, water temperature, and relative swimming speed. Retaining the established framework of Grøttum and Sigholt (1998), we refined the model through an improved coefficient estimation approach and a methodologically rigorous dataset derived from group swim tunnel respirometry measurements on 718 fish across seven experiments. Model coefficients were re-estimated using log-linear regression fitted via nonlinear mixed-effects, substantially improving parameterisation and yielding a model that explains 80% of the observed variation in MO_2_:${MO}_{2}=79.7{W}^{-0.14}1.0{4}^{T}1.{63}^{U}$, where MO_2_ is oxygen consumption rate (mg O_2_ kg^− 1^ h^− 1^), W is body weight (kg), T is water temperature (°C), and U is relative swimming speed (body lengths s^− 1^). Our model delivers reliable estimates of Atlantic salmon oxygen demand across relevant farming conditions (0.2–3.4 kg, 3–18 °C, 0.3–2.8 body lengths s^− 1^). With broad utility in both research and industry, our model supports the development of more precise, data-driven strategies for modern salmon aquaculture.

## Introduction

Atlantic salmon (*Salmo salar*) aquaculture is evolving rapidly, with production units becoming larger and transitioning towards offshore cages, semi-closed or closed systems, and land-based tanks^1,2^—all operated at stocking densities that create enormous oxygen demands^3–6^. In practice, most salmon are still farmed in open sea cages, where maintaining a suitable oxygen environment remains one of the most pressing challenges. Dissolved oxygen is a critical determinant of salmon welfare^7,8^. Even brief periods of low oxygen can compromise health^9,10^ and depress appetite^11,12^, while severe deficits can trigger catastrophic mortality events^13,14^. These risks highlight the need for proactive oxygen management to safeguard salmon welfare in farming systems.

Three-dimensional (3D) digital simulations that integrate bioenergetic and hydrodynamic models with environmental data are emerging as powerful tools for this purpose^15^. By capturing how environmental and endogenous factors interact to shape oxygen availability, these simulations provide valuable insights for farm management. When robust, these simulations could offer transformative industry applications such as forecasting oxygen levels to prevent hypoxia-related welfare issues^16,17^, evaluating the suitability of new aquaculture sites^18,19^, and prototyping novel production designs^20,21^. Advancing digital simulations should be a priority in aquaculture research, as they embody the replacement principle, a cornerstone of humane experimental techniques^22^.

Replicating the oxygen dynamics of aquaculture systems is highly complex, driven by broad variation in oxygen demand^23^ and the dynamic spatial distribution of salmon in farmed populations^24^. Fortunately, most of the variation in oxygen demand can be explained by three key factors: body weight, water temperature, and swimming activity^25–28^. The success of 3D digital simulations therefore depends on reliable estimates of salmon oxygen consumption rate (MO_2_; mg O_2_ kg^− 1^ h^− 1^), yet prior empirical models remain limited in accuracy. Although many MO_2_ models have been proposed^28–30^, they vary considerably in form and predictive power due to the complexities of measuring MO_2_ across scenarios^23,31,32^. As a result, current models often fail to predict oxygen demand with sufficient accuracy^28^. Indeed, Berntsson et al.^23^ noted that the nearly 30-year-old model of Grøttum and Sigholt^29^ remains the most widely applied in both research and industry, despite its clear limitations. This continued reliance on outdated models underscores the urgent need for a modern alternative to support future oxygen management in salmon aquaculture. A recently developed simulation by Alver et al.^16,20,21^ has made significant progress in digitally replicating oxygen dynamics in sea cage environments. However, their current approach^21^ still approximates MO_2_ using a modified version of the original Grøttum and Sigholt^29^ model, described in Eq. 1 below:

1 $$\:{MO}_{2}=61.6{W}^{-0.33}1.0{3}^{T}1.7{9}^{U}$$

where MO_2_ is oxygen consumption rate (mg O_2_ kg^− 1^ h^− 1^), W is body weight (kg), T is water temperature (°C), and U is relative swimming speed (body lengths [BL] s^− 1^).

This core formulation defines baseline MO_2_ for Atlantic salmon and serves as a foundational component within digital simulation frameworks^16,20,21^, enabling the modular integration of additional farm-relevant processes as simulation complexity increases. While the structure of Grøttum and Sigholt’s^29^ empirical model is still valid^30^, the approximation likely underestimates the MO_2_ of modern farmed salmon, as reflected by group-level MO_2_ established by Hvas & Oppedal^33^. To address this pragmatically, Alver et al.^16,20^ increased their MO_2_ estimates by 30%, and in their most recent model, applied an additional 20% increase to improve site-specific model agreement^21^. These successive ad-hoc adjustments highlight the need for a revised MO_2_ model tailored to contemporary aquaculture.

Grøttum and Sigholt’s^29^ original model (Eq. 1) was derived from individual respirometry measurements on six third-generation selectively bred salmon^34^. These six fish spanned 1.1–2.1 kg and had their MO_2_ measured at water temperatures of 5–15 °C and relative swimming speeds of 0.5–1.5 body lengths (BL) s^− 1^. Some of these MO_2_ measurements were preceded by abrupt 5 °C temperature shifts and short 10–15 h acclimation periods. These methodological limitations—small sample size, narrow data ranges, individual rather than group measurements, limited thermal acclimation, and outdated genetics—constrain its relevance to today’s farmed salmon populations.

**Fig. 1:**
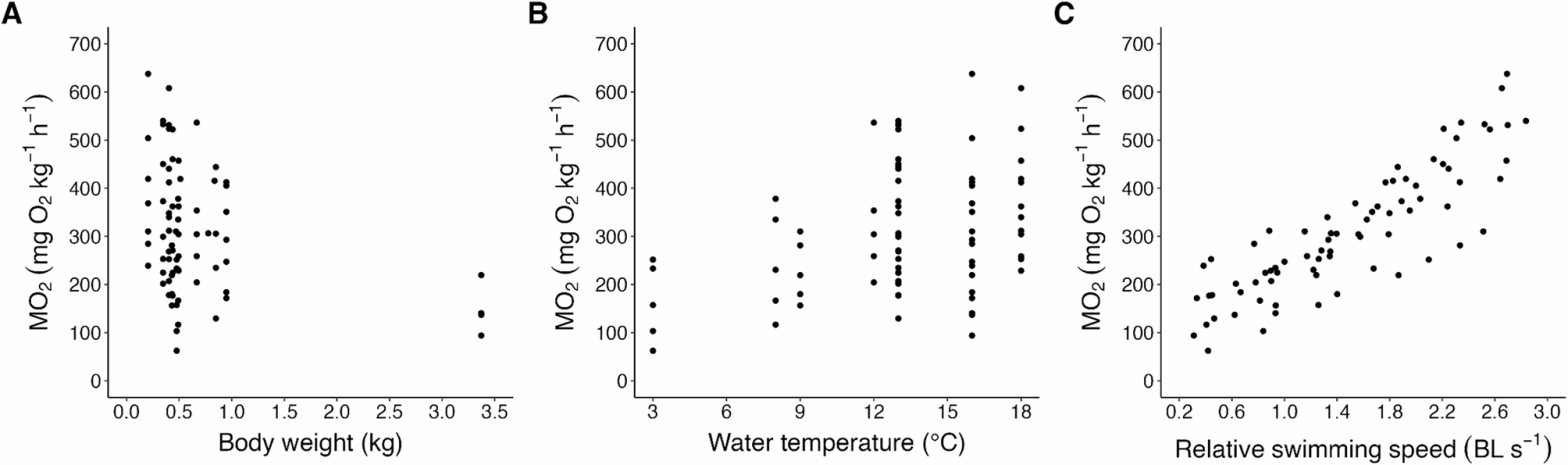
Overview of refined dataset displaying oxygen consumption rate (MO_2_; mg O_2_ kg^-1^ h^-1^) across the three primary parameters: (**A**) body weight (kg), (**B**) water temperature (°C), and (**C**) relative swimming speed (body lengths [BL] s^-1^).

To overcome these limitations, we compiled a new dataset which aggregates group swim tunnel respirometry data from 718 fish across seven studies (Table 1). All of these studies were conducted using the large Brett-type swim tunnel developed by the Norwegian Institute of Marine Research for testing swimming performance in groups of larger-sized salmonids^35^. This system was later modified to measure group-level MO_2_^36^, providing a method of respirometry that is equally reliable to conventional individual measurements^37^, while yielding data more representative of commercial farming conditions^35^. Furthermore, a recent study confirmed that the swimming performance of tank-reared Atlantic salmon matches that of sea cage-reared fish, supporting the dataset’s relevance for both production systems^38^. Importantly, all studies used a modern domesticated strain (AquaGen) that has undergone over ten generations of selective breeding for improved growth and survival^34,39^, resulting in marked physiological differences from earlier strains used in the 1990s^40,41^. Finally, our refined dataset (Fig. 1) spans broader data ranges than those of Grøttum and Sigholt^29^, covering fish sizes from 0.2 to 3.38 kg, water temperatures of 3–18 °C after several weeks of thermal acclimation, and relative swimming speeds of 0.31–2.84 BL s^− 1^.

Leveraging this extensive dataset of group swim tunnel respirometry measurements (Table 1; Fig. 1), we developed a refined fundamental model for oxygen consumption of Atlantic salmon. Retaining the framework of Grøttum and Sigholt^29^, we refined model coefficients using log-linear regression fitted via nonlinear mixed-effects. With demonstrably greater predictive accuracy than previous empirical approaches, our model provides a robust and timely upgrade for estimating salmon oxygen demand—equipping aquaculture scientists, engineers, and farm operators with a powerful tool for research and industry applications.

## Methods

### Criteria for data inclusion

To compile the dataset for this study, we included all published swim tunnel respirometry experiments conducted by the Norwegian Institute of Marine Research using the large Brett-type swim tunnel respirometer originally described by Hvas, Folkedal et al.^36^. In total, seven studies conducted between 2017 and 2022 were included (Table 1). This swim tunnel accommodates groups of post-smolt Atlantic salmon swimming simultaneously, enabling direct and accurate measurement of group-level MO_2_ under controlled conditions^37^. In said experiments, treatment groups were defined based on variables such as water temperature, body weight, hypoxia, salinity, swimming speed, health status, and fasting state. When sourcing data from each experiment, only trials that met a consistent set of criteria were included. These criteria were selected to reflect typical operating conditions encountered in aquaculture systems. All measurements were obtained from either critical swimming speed or sustained swimming speed tests. To ensure environmental comparability, we included only trials conducted in full-strength seawater, normoxia, and with standardised temperature acclimation protocols: fish were held at the target experimental temperature for a minimum of three weeks, followed by overnight acclimation in the swim tunnel before experimentation. We deliberately selected fish that were fed during the entirety of their temperature acclimation period and only fasted during their overnight acclimation period in the swim tunnel so that their MO_2_ reflected a fed-state. Data was extracted exclusively from treatment groups in which fish were deemed healthy. Additionally, all Atlantic salmon used across experiments originated from a modern domesticated strain to ensure both genetic consistency and industry relevance (AquaGen strain). These selection criteria ensured that all data points were methodologically consistent and biologically representative of standard operations for commercial salmon aquaculture. The selected treatment groups featured 2–31 Atlantic salmon swimming simultaneously, resulting in stocking densities within the range typically observed (~ 15–35 kg m^− 3^) in salmon aquaculture systems^24,42^. These stocking densities provided sufficient resolution to capture differences in MO_2_ across gradients of the three primary parameters: body weight, water temperature, and swimming speed.

For all treatment groups that met our selection criteria, we identified each unique combination of body weight, water temperature, and relative swimming speed, then aggregated MO_2_ data across the affiliated 2–6 replicate trials within each study. The number of treatment groups and replicate trials varied among studies depending on experimental design. Within a given replicate trial, water temperature and swimming speed were consistent for all fish, whereas body size varied among individuals within a narrow range corresponding to their assigned size class. Accordingly, group-average body weights and fork lengths—initially calculated for each replicate trial—were further averaged across affiliated replicates to yield single representative values for each treatment group. The replicate-aggregated mean MO_2_ for each unique combination of the three primary parameters within each treatment group constituted one data entry. Compiling all eligible treatment groups across all experiments yielded a complete dataset of 96 data entries, spanning biological ranges of 0.2–3.38 kg, 3–23 °C, and 0.31–3.16 BL s^− 1^. To maximise model accuracy and ensure adherence to model assumptions, we omitted 20 data entries (as detailed in Section “Modelling framework and assumptions”.) to form a refined dataset of 76 entries (Table 1; Fig. 1).

### Ethics statement

This study involved no primary animal experimentation. All analyses were based on data from previously published experiments which were approved by the Norwegian Food Safety Authority and conducted in accordance with the Norwegian Animal Welfare Act (LOV-2009-06-19-97) and ARRIVE guidelines.

### Modelling framework and assumptions

To describe the group-level MO_2_ of Atlantic salmon, we adopted a previously published model structure developed by Grøttum & Sigholt^29^, which expresses MO_2_ as a function of three primary parameters: body weight, water temperature, and relative swimming speed. The model’s functional form is based on established physiological principles, as outlined by Grøttum & Sigholt^29^. To summarise, the relationship between body weight and MO_2_ follows a classic allometric scaling equation^43–47^. Meanwhile, the influence of temperature on MO_2_ follows an exponential function^25,43,48^ similar to equations describing thermal dependence of biochemical reaction rates^49–51^. Finally, relative swimming speed can be described by an exponential function^32,52^. Mechanistically, temperature dependence is best described by an Arrhenius-type relationship^49–51^ while swimming speed dependence is best described by hydrodynamic theory, which predicts a power function because drag scales with the square of swimming speed and the power to overcome it scales with the cube^53,54^. Nonetheless, an exponential curve for both temperature and swimming speed dependence consistently fits the biological ranges represented in our dataset. Given this practical equivalence, we retained the original empirical structure proposed by Grøttum and Sigholt^29^ to enable direct comparison between our updated coefficients and those of the previous model.

Together, these three independent components are aggregated to estimate MO_2_ using the general functional form described by Eq. 2 below:

2 $$\:{MO}_{2}=a{W}^{b}{c}^{T}{d}^{U}$$

where MO_2_ is oxygen consumption rate (mg O_2_ kg^− 1^ h^− 1^), W is body weight (kg), T is water temperature (°C), U is relative swimming speed (body lengths s^− 1^), while a, b, c, and d, are model coefficients to be estimated.

Recent experiments with farmed Atlantic salmon confirm that these mathematical trends accurately reflect observed data within certain constraints. The predicted shapes align with physiological observations across the full body weight range, but not across the full range of water temperatures and relative swimming speeds. At higher temperatures and relative swimming speeds, aerobic metabolism plateaus due to performance limits^36,55^, thus including measurements within these plateaus would lower overall MO_2_ predictions, compromising model validity across the entire biological range. Capturing such plateau effects would require replacing the exponential functions for temperature and swimming speed with sigmoidal functions, doubling the number of model coefficients from four to eight and severely compromising practical applicability. To avoid these downsides, we deliberately constrained our dataset to lower biological limits, ensuring the data meets the assumptions of the modelling framework while maximising predictive precision.

We restricted our dataset by excluding one treatment group held at 23 °C (rationale explained in Section “Extrapolation of water temperature”.). In addition, we omitted relative swimming speed values at 100% of the critical swimming speed for each treatment group (rationale explained in Section “Extrapolation of relative swimming speed”.), resulting in maximum swim values between 80 and 90% of group-specific critical swimming speeds. We believe these refinements balance model precision with retention of the largest possible dataset. After these omissions, the dataset was reduced from 96 to 76 entries, yielding a refined dataset with biological ranges of 0.2–3.38 kg, 3–18 °C and 0.31–2.84 BL s^− 1^ (Table 1; Fig. 1).

### Overview of refined dataset

**Table 1:** Summary of experiments included in the refined dataset. Asterisks (*) denote treatment variables within each experiment. Experiments are listed in order of their proportional contribution to the dataset based on the number of data entries. Body weight values represent the mean across affiliated replicates to yield a representative value for each treatment group. BL = body lengths; n = number of individuals.

Study	Primary parameters			Experimental representation		
	Body weight (kg)	Water temperature (°C)	Relative swimming speed (BL s^− 1^)	Group size per trial (*n*)	Total fish analysed (*n*)	Proportion of dataset (%)
Hvas, Folkedal, et al. (2017)^36^	0.452	3 (*) 8 13 18	0.4–2.66	10	240	28.96%
Oldham et al. (2019)^55^	3.372 (*) 0.949 0.205	16	0.31–2.70	2 7–10 27–31	120	23.68%
Hvas (2022)^56^	0.477	9 (*) 18	0.89–2.69	8	96	14.47%
Hvas, Karlsbakk, et al. (2017)^57^	0.347	13	0.63–2.84	15	120	10.53%
Hvas & Oppedal (2017)^33^	0.821	13	0.46–1.87	8	72	7.89%
Hvas et al. (2018)^58^	0.403	13	0.44–2.70	10	40	7.89%
Hvas et al. (2021)^59^	0.667	12	0.78–2.35	10	30	6.58%

To evaluate the distribution of the data used for model development, MO_2_ values were plotted against body weight, water temperature, and relative swimming speed (Fig. 1). Water temperature and relative swimming speed showed good coverage across their respective ranges, supporting reliable coefficient estimation. However, body weight was sparsely represented above 1 kg, with only 5.3% of our refined data entries covering this range. This left a clear gap between 1 and 3.3 kg that may reduce model accuracy within this size class. Consequently, our model is expected to provide the most reliable MO_2_ estimates for fish weighing 0.205–0.949 kg and ~ 3.38 kg.

### Formulation of revised model

Using R version 4.5.1^60^ and our refined dataset, we estimated model coefficients with log-linear regression fitted via nonlinear mixed-effects, following a similar two-stage framework employed by Macnaughton et al.^30^, who derived a standard metabolic rate model for Atlantic salmon. In our first step, we log-transformed both the MO_2_ and body weight to linearise the multiplicative model structure. A linear regression model was then fitted with log-transformed MO_2_ as the response and log-transformed body weight, water temperature, and relative swimming speed as predictor variables. The resulting regression coefficients provided biologically interpretable starting values: the intercept (log-a) was exponentiated to obtain a, the coefficient of log body weight represented b, and the coefficients of water temperature and relative swimming speed were exponentiated to derive initial values for c and d, respectively. In our second step, these initial estimates were then used to fit a nonlinear mixed-effects model directly to the untransformed data; study ID was included as a random effect using the nlme() function from the ‘nlme’ package^61^. All two-way interactions between primary parameters (body weight, water temperature, and relative swimming speed) were evaluated using mixed-effects nonlinear models; no interaction term was retained as none was statistically supported over the others, and no independent biological rationale existed to justify selecting one above the rest. The final fitted model returned refined estimates of the coefficients a, b, c, and d, describing the multiplicative effects of body weight, water temperature, and relative swimming speed on MO_2_.

## Results

### Revised model coefficients and validations

The log-linear regression fitted via nonlinear mixed-effects showed the strongest performance, with adjusted R^2^ = 0.80 and root mean square error (RMSE) = 56.39, outperforming the original model by Grøttum and Sigholt^29^ which had an adjusted R^2^ = 0.68 and RMSE = 71.50 (Table 2).

**Table 2:** Summary of approaches, estimated model coefficients ± standard errors (SE), and validations including adjusted R^2^ and root mean square error (RMSE).

Model description		Model coefficients (± SE)				Validation	
Approach	Dataset	a	b	c	d	Adjusted *R*^2^	RMSE
Log-linear regression fitted via nonlinear mixed-effects	Refined	79.70 ± 6.27	-0.14 ± 0.04	1.04 ± 0.004	1.63 ± 0.03	0.80	56.39
Multiple nonlinear regression analysis	Grøttum and Sigholt (1998) [29]	61.6 ± 6.6	-0.33 ± 0.11	1.03 ± 0.10	1.79 ± 0.10	0.68	71.50

**Fig. 2:**
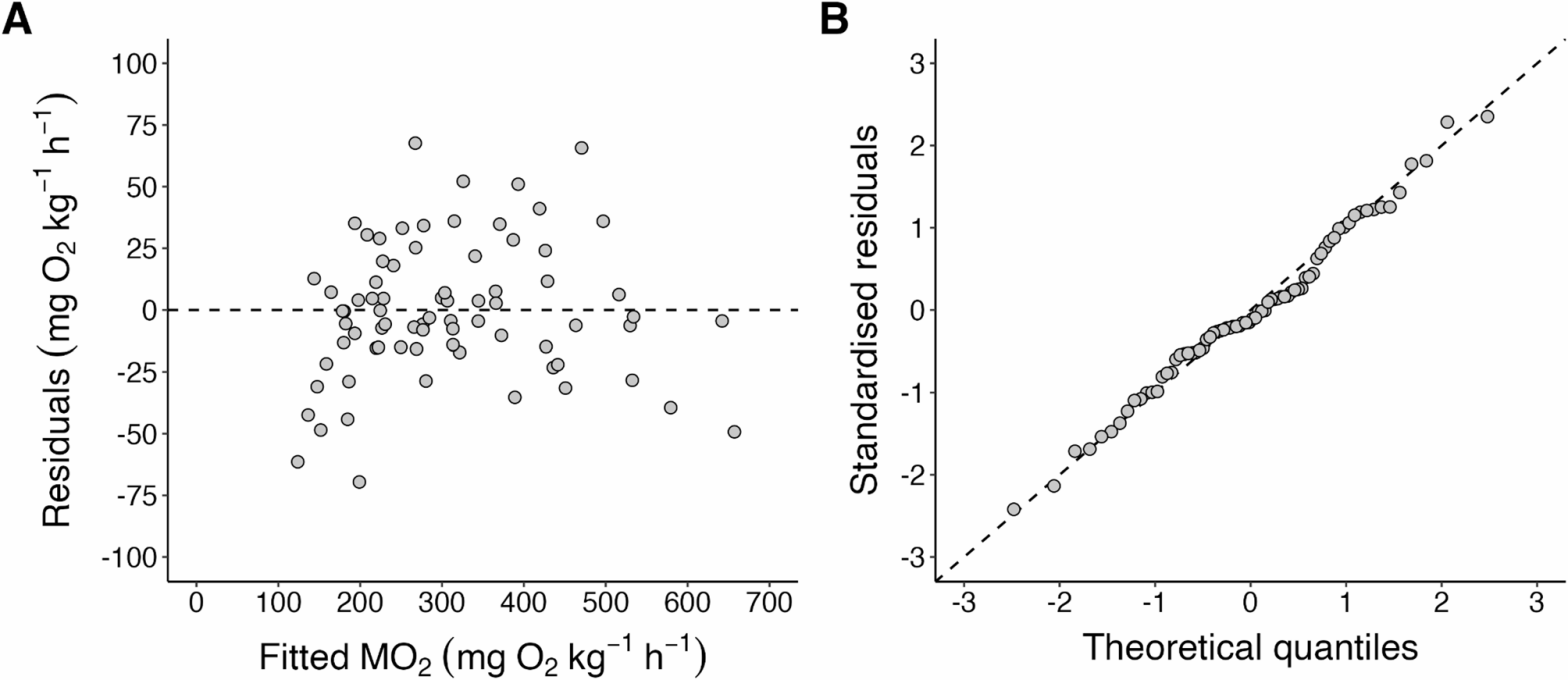
Oxygen consumption rate (MO_2_; mg O_2_ kg^-1^ h^-1^) model diagnostic plots computed via the nonlinear mixed-effects model fit to the entire refined dataset. (**A**) Residuals plotted against fitted MO_2_ values; the dashed line denotes zero. (**B**) Normal Q-Q plot of standardised residuals against theoretical quantiles; the dashed line represents the expected normal distribution.

Log-linear regression fitted via nonlinear mixed-effects yielded model coefficients that were well constrained, with narrow 95% confidence intervals (a: 67.50–91.89; b: −0.19– −0.09; c: 1.03–1.05; d: 1.57–1.68). Residual diagnostics indicated that the model assumptions were adequately met (Fig. 2). Residuals were randomly distributed around zero across the range of fitted MO_2_ values, showing no clear systematic trend and indicating that the model broadly captures the primary structure of the data without substantial bias (Fig. 2A). Some heteroscedasticity is evident at higher fitted values, reflecting greater absolute variability at elevated MO_2_ levels. The Q–Q plot (Fig. 2B) shows that the standardized residuals closely follow the expected normal distribution, with only minor deviations at the distribution tails.

Formulated using log-linear regression fitted via nonlinear mixed-effects (adjusted R^2^ = 0.80), our model is as follows:

3 $$\:{MO}_{2}=79.7{W}^{-0.14}1.0{4}^{T}1.{63}^{U}$$

where MO_2_ is oxygen consumption rate (mg O_2_ kg^− 1^ h^− 1^), W is body weight (kg), T is water temperature (°C), and U is relative swimming speed (body lengths s^− 1^).

### Visualisation of revised model

**Fig. 3:**
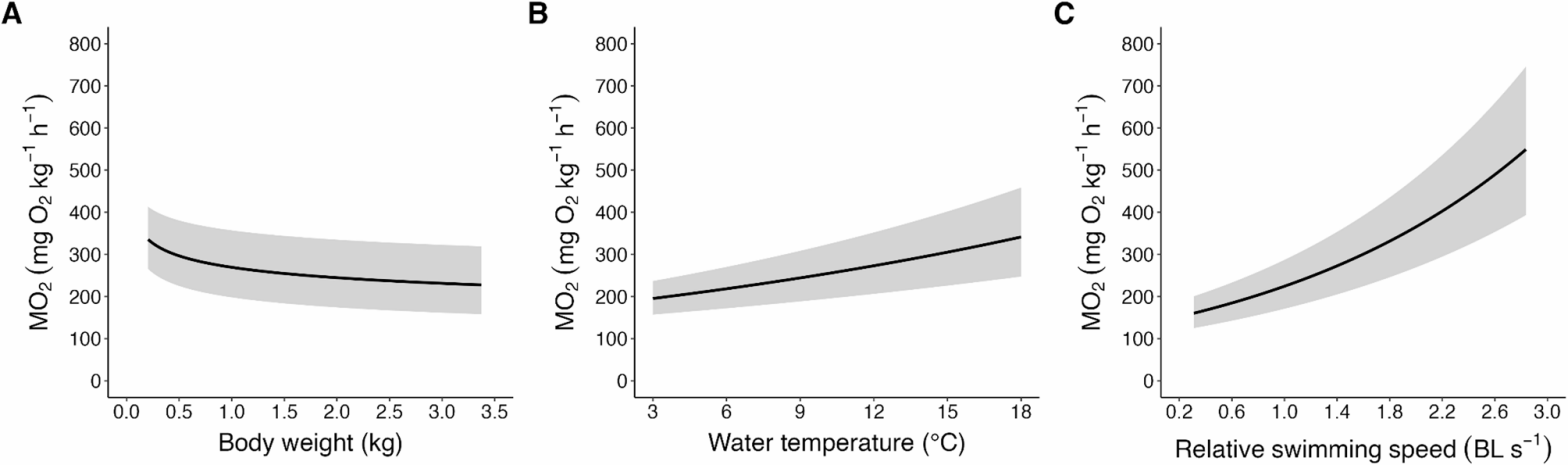
Modelled predictions of oxygen consumption rate (MO_2_; mg O_2_ kg^-1^ h^-1^) across the three primary parameters based on the nonlinear mixed-effects model fit to the refined dataset. (**A**) Body weight (kg), (**B**) water temperature (°C), and (**C**) relative swimming speed (body lengths [BL] s^-1^) varied across their observed ranges while the other parameters were held at their mean values. Shaded regions represent 95% confidence intervals.

**Fig. 4:**
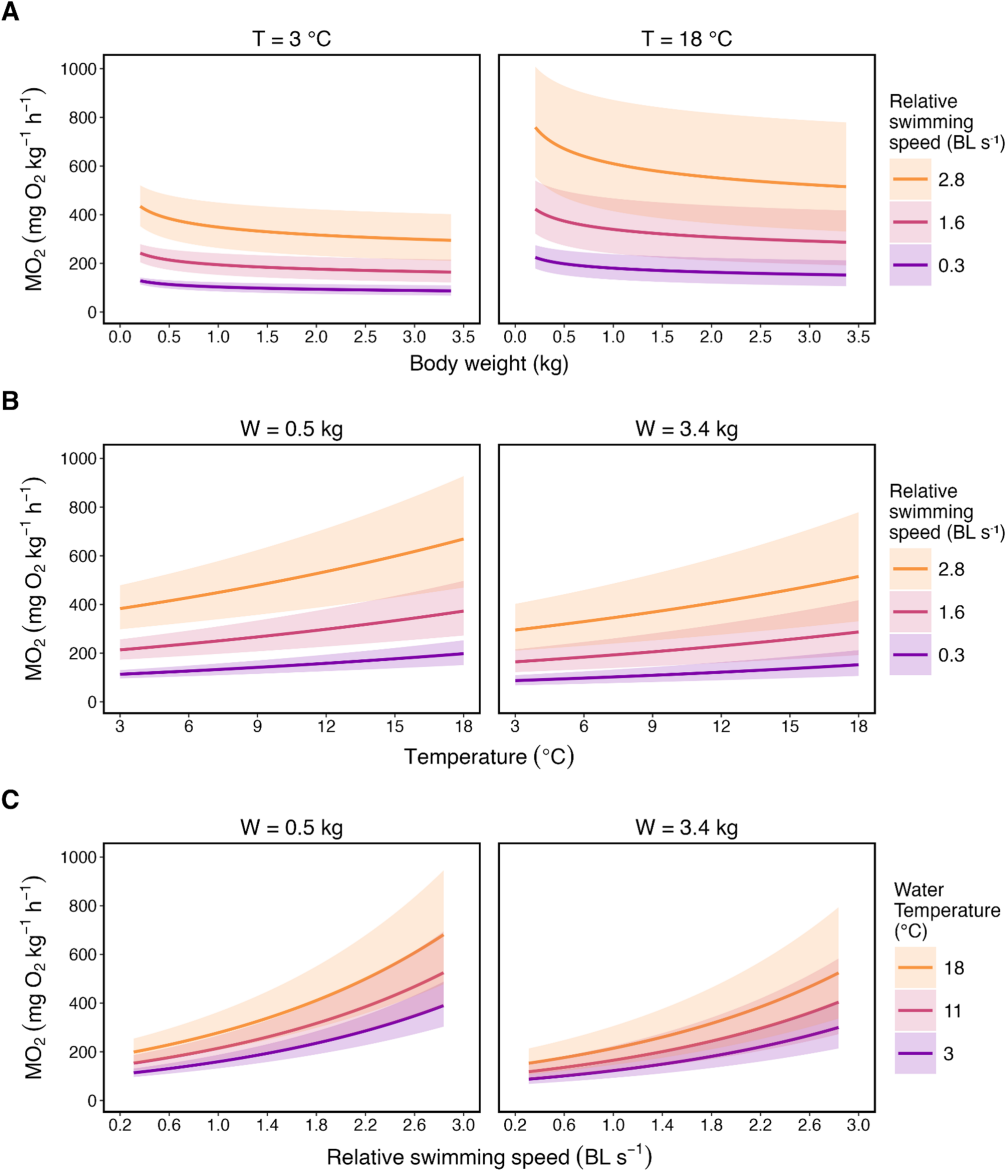
Modelled predictions of oxygen consumption rate (MO_2_; mg O_2_ kg^-1^ h^-1^) across the three primary parameters based on the nonlinear mixed-effects model fit to the refined dataset. (**A**) MO_2_ as a function of body weight (kg), faceted by model margins of water temperature (3 and 18 °C) and coloured by relative swimming speed range (0.3, 1.6, and 2.8 body lengths [BL] s^-1^). (**B**) MO_2_ as a function of water temperature (°C), faceted by representative body weights (0.5 and 3.4 kg) and coloured by relative swimming speed range (0.3, 1.6, and 2.8 BL s^-1^). (**C**) MO_2_ as a function of relative swimming speed (BL s^-1^), faceted by representative body weights (0.5 and 3.4 kg) and coloured by water temperature range (3, 11, and 18 °C). Shaded regions represent 95% confidence intervals. W = body weight; T = water temperature.

**Fig. 5:**
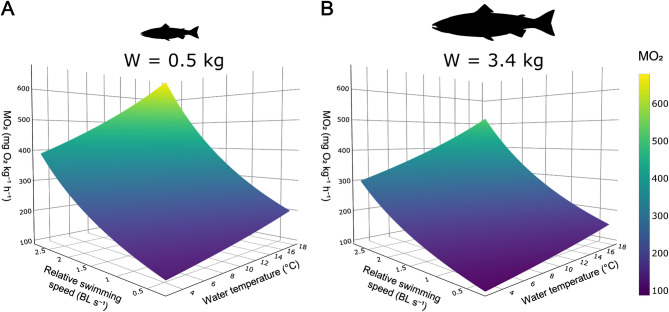
Three-dimensional surface contours illustrating oxygen consumption rate (MO_2_; mg O_2_ kg^-1^ h^-1^; z-axis) as a function of relative swimming speed (U; body lengths [BL] s^-1^; y-axis) and water temperature (T; °C; x-axis). Results are displayed for two body weights (W; kg): (**A**) 0.5 kg and (**B**) 3.4 kg, selected as the most representative weights of our refined dataset (Table 1; Fig. 1). Surface contours are generated from our model developed using log-linear regression fitted via nonlinear mixed-effects (Eq. 3): MO_2_ = 79.7 W^-0.14^1.04^T^1.63^U^.

To evaluate the shape and behaviour of our model, we generated a series of visualisations to examine both average model performance (Fig. 3) and predictions at the extremes of the biological ranges within our refined dataset (Fig. 4). Two-dimensional (2D) plots highlight the effects of each primary parameter individually, while a 3D surface integrates these effects into a single representation of the full response surface (Fig. 5).

#### 2D visualisation of revised model

Using the coefficients from the nonlinear mixed-effects model described in Section “Formulation of revised model”., we plotted predictions of MO_2_ along each primary parameter with 95% confidence intervals. Each focal parameter varied across its observed range while the other parameters were held at their mean values (Fig. 3).

To further illustrate model behaviour across realistic biological scenarios, we generated faceted 2D plots that combine interacting parameters (Fig. 4), allowing us to understand how the model behaves in a variety of scenarios. To achieve this for each focal parameter, we faceted and/or colour coded across model margins for body weights (0.5 and 3.4 kg), water temperature (3, 11, and 18 °C) and relative swimming speeds (0.3, 1.6, and 2.8 BL s^− 1^). Specifically, the mean weight of 0.51 kg was selected to represent fish in the 0.205–0.949 kg range, while 3.37 kg represents fish from the largest treatment groups, reflecting both early and late stages of salmon production.

#### 3D visualisation of revised model

To integrate the effects included in our model (Eq. 3) into a comprehensive response surface, we visualised predicted MO_2_ in three dimensions for two representative body weights from the refined dataset (Fig. 5).

These surfaces from Fig. 5 confirm the physiological patterns observed in the 2D plots (Figs. 3 and 4). As expected from the exponential terms of the model, MO_2_ increases with both relative swimming speed and water temperature, each tracing the early phase of an upward exponential curve in each dimension. When combined in three dimensions, these patterns produce a smooth and continuous response surface. Across all conditions, 0.51 kg fish exhibit higher mass-specific MO_2_ than 3.37 kg fish, consistent with established allometric principles of metabolic scaling^45,47^ and previous findings in Atlantic salmon^55^.

## Discussion

Our refined fundamental model for oxygen consumption of Atlantic salmon represents a substantial advance over previous empirical models. It spans an extensive range of metabolic rates (60–700 mg O_2_ kg^− 1^ h^− 1^), fish sizes (0.2–3.38 kg), water temperatures (3–18 °C), and relative swimming speeds (0.31–2.84 BL s^− 1^), capturing the diversity of conditions in modern salmon aquaculture. This core formulation defines baseline MO_2_ for Atlantic salmon and serves as a foundational component within digital simulation frameworks, enabling the modular integration of additional farm-relevant processes as simulation complexity increases. Built from a contemporary and methodologically rigorous dataset, our model provides high confidence in its predictive accuracy (adjusted R^2^ = 0.80). By relying on readily measurable parameters, our model offers practical utility for both research and aquaculture operations. In the following sections, we detail why our model delivers more reliable estimates than its predecessors and outline best practices for its effective application.

### Interpretation of revised model coefficients

To conceptually understand how our model (Eq. 3) differs from the original (Eq. 1) proposed by Grøttum and Sigholt^29^, it is useful to examine how each coefficient influences the shape of the MO_2_ prediction curve. Coefficient a, the intercept, increased from 61.6 to 79.7, raising the entire MO_2_ curve by 29.4%. This is a reassuring revision given Alver et al.’s successive increases to MO_2_ predictions—first by 30%^16,20^, then by an additional 20% in their latest model^21^.

Coefficient b, the body weight exponent, increased by 58.0% from -0.33 to -0.14, ultimately flattening the negative exponential curve compared to Grøttum and Sigholt’s model^29^. This flattening effect results in lower MO_2_ predictions for fish below 1 kg and higher predictions for those above 1 kg. Following classic allometric theory, we would expect to find an absolute metabolic rate scaling exponent of 0.75^45,47^ or a mass-specific metabolic rate scaling exponent of -0.25^43^. Our mass-specific metabolic rate scaling exponent of -0.14 is noticeably smaller than Grøttum and Sigholt’s^29^ estimation of -0.33 and the classical predictions of -0.25, but this was not unexpected. Among fishes, mass-specific resting metabolic rate typically scales with body mass at an exponent around − 0.21^25^. However, as activity level increases, metabolic scaling can become nearly isometric with body mass, likely due to volume-related muscular power production^62^. This pattern has been observed in several fish species, including farmed Atlantic salmon, whereby mass-specific scaling exponents during active exercise are closer to 0 than at rest^55,63^. Since our dataset reflects slow, moderate, and strenuous swimming speeds rather than resting conditions, we expected the scaling exponent to be closer to 0. Our observed value of -0.14 is therefore consistent with recent allometric theory, which predicts values around − 0.08 for active ectothermic animals^63^.

The temperature coefficient c changed from 1.03 to 1.04, slightly increasing the steepness of the MO_2_ rise with temperature. This value can be used to derive the Q_10_ coefficient, which describes the multiplicative change in metabolic rate associated with a 10 °C temperature increase. Although the coefficient c disparity is marginal, 1.03 corresponds to a Q_10_ of 1.34 while 1.04 corresponds to a Q_10_ of 1.45. This means Grøttum and Sigholt’s^29^ MO_2_ predictions increase by 34% for every 10 °C rise in water temperature, while our revised MO_2_ predictions increase by 45%, equivalent to a 32% greater temperature sensitivity spanning 10 °C. We hypothesise that this difference reflects the longer thermal acclimation periods (≥3 weeks) used in our dataset. Grøttum and Sigholt^29^ performed MO_2_ measurements after abrupt 5 °C temperature shifts and short 10–15 h acclimation periods; fish subjected to these conditions may experience acute stress, potentially confounding MO_2_ measurements and compressing apparent temperature sensitivity. For ectothermic fish like Atlantic salmon—whose body temperature doesn’t change during exercise—resting MO_2_ between 3 and 23 °C yields a Q_10_ of 2.2, whereas active MO_2_ at ~ 0.4–2.6 BL s^− 1^ over the same temperature range yields a Q_10_ of 1.63^36^, which is much more similar to our model’s Q_10_ of 1.45. This points to a broader trend whereby temperature has a weaker proportional effect on MO_2_ when fish are swimming than when they are resting. This observation is intuitive: as fish increase swimming effort, a larger fraction of their metabolic scope is already dedicated to locomotion, leaving less relative influence for temperature on MO_2_. Thus, similar to the downward adjustment of the mass-scaling coefficient b under active conditions, the temperature-scaling coefficient c is also reduced. Both effects highlight how exercise attenuates the influence of size and temperature on MO_2_ relative to rest.

Finally, the relative swimming speed coefficient d decreased by 9.1%, from 1.79 to 1.63, reducing the steepness of the MO_2_ rise with relative swimming speed. This decrease is most likely due to the increased range of our dataset which extends to relative swimming speeds of 2.84 BL s^− 1^, much faster than the 1.5 BL s^− 1^ covered by Grøttum & Sigholt^29^. As relative swimming speed increases, aerobic metabolism begins to plateau^27,36^ as performance limitations are approached and fish begin to swim anaerobically^64,65^. Although we truncated our dataset to remove the highest relative swimming speeds at 100% critical swimming speed, the observed reduction in our relative swimming speed coefficient d is logical and expected.

Taken together, our adjustment to coefficient a resulted in a net increase in predicted MO_2_, coefficient b flattens MO_2_ predictions across body weights, coefficient c marginally increases the steepness of the MO_2_ rise with temperature, and coefficient d makes the positive relationship between MO_2_ and relative swimming speed less steep. These interacting revisions have resulted in improved overall predictive precision of our model (adjusted R^2^ = 0.80) compared to the model proposed by Grøttum and Sigholt^29^(adjusted R^2^ = 0.68).

### Best practices for applying our model

We expect our model to perform reliably across both tank and sea-cage production systems^38^, with strongest performance within the biological conditions it was established with: 0.2–3.38 kg, 3–18 °C, 0.31–2.84 BL s^− 1^, and normoxia. Below, we consider extrapolation beyond these limits where observations may begin to depart from model assumptions. Extrapolation should seldom be necessary since the established ranges already encompass most commercial sea-cage farming conditions. Farmed Atlantic salmon in Norway are typically transferred to sea at ~ 0.1 kg^66^ with average harvest weights reaching 4.25 kg^67^. Across major salmon farming regions, seasonal water temperatures at 3 m depths span 0.1–23.1 °C^66,68,69^, while deeper waters in stratified sites are typically less extreme. Within this range, salmon actively select depths near their 16–17 °C thermal preference while avoiding water ≥ 18 °C^24^, as chronic exposure to extremes can compromise welfare^7,8,36^. Water current speeds within sea cages are often low, with prevailing water currents ranging from 0 to 20 cm s^− 1^ in coastal sites^18^ and 5–9 cm s^− 1^ in fjords^4^, corresponding to voluntary swimming speeds of ~ 0.6–1.4 BL s^− 1^ during the day and < 0.5 BL s^− 1^ at night^24,70^. Broader datasets similarly report relative swimming speeds of 0.2–2.8 BL s^− 1^^24,71–76^. Finally, marine farms typically maintain conditions above the limiting oxygen saturation^10,12^ throughout the sea cage environment^4,5,24^.

#### Extrapolation of body weight

Regarding body weight, extrapolation beyond these bounds towards the ~ 0.1 kg sea transfer^66^ and the typical harvest size of 4.25 kg^67^ should remain accurate. The allometric scaling of MO_2_ with body weight should hold across many orders of magnitude, underpinned by well-established mechanistic principles of resource distribution networks^77–80^. This provides a predictable basis for extending our MO_2_ model to smaller and larger sizes. Indeed, considering that Atlantic salmon can reach up to 49.44 kg in the wild^81^—orders of magnitude above aquaculture-relevant sizes—extrapolation towards the industry average harvest weight of 4.25 kg is modest and likely reliable, also extending to the sites targeting larger fish between 4.8 and 6 kg^66^.

#### Extrapolation of water temperature

Regarding water temperature, extrapolation from our model’s lower margin of 3 °C towards near-freezing conditions (e.g., 0.1 °C;^68)^ appears valid. Empirical studies show minimal differences in MO_2_ between 0 and 3 °C, matching the shallow slope predicted by an exponential function^82,83^. As a cold-adapted, winter-active species, Atlantic salmon also maintain normal MO_2_ at near-freezing temperatures without evidence of abnormal suppression or elevation^82–84^. Therefore, MO_2_ scales smoothly down to 0 °C in line with exponential predictions. Atlantic salmon cannot survive below 0 °C due to plasma freezing^85^, so extrapolation beyond this point is not appropriate.

We caution against extrapolating our model beyond its upper thermal margin of 18 °C. MO_2_ up to 18 °C tends to follow an exponential temperature dependence^10^. However, as shown by MO_2_ measurements at 23 °C in our dataset, metabolic rate plateaus as salmon approach their upper thermal tolerance rather than continuing to rise exponentially^36^, justifying our decision to exclude the 23 °C treatment group from our refined dataset. This plateauing in temperature sensitivity is reflected in Q_10_ values: for standard metabolic rate, Q_10_ declines from 3.1 between 3 and 13 °C to 1.6 between 13 and 23 °C, while for active metabolic rate (measured across ~ 0.4–2.6 BL s^− 1^) Q_10_ declines from 1.91 to 1.39 across the same ranges^36^. Although salmon can still elevate both standard and active metabolic rates at 23–24 °C, the MO_2_ plateau is more pronounced in active metabolic rate^36,86^. Recent experiments further demonstrate ~ 3.74 kg Atlantic salmon were unable to increase their max MO_2_ between 9 and 19 °C^87^. Consequently, extrapolations above 18 °C are expected to overestimate MO_2_, particularly when fish are large and relative swimming speeds are fast. However, values up to 23 °C are likely only minorly inflated and still functionally informative, particularly for smaller fish and at slower swimming speeds. Since Atlantic salmon MO_2_ likely peaks just below the lethal threshold, which occurs at chronic seawater exposure to 21–23 °C^36,88^, we recommend capping extrapolations at ~ 23 °C and treating any extrapolations above 18 °C with caution.

#### Extrapolation of relative swimming speed

Regarding relative swimming speed, we recommend extrapolating our model down to zero, as a substantial body of research confirms the exponential rise in MO_2_ from rest to moderate relative swimming speeds in salmonids^27,52,89^. A methodological study demonstrated that MO_2_, expressed as an exponential function of relative swimming speed, can be extrapolated to zero using active metabolic rate from group swim tunnel respirometry, yielding accurate estimates of individual standard metabolic rate obtained from static respirometers^37^. However, this approach has limited relevance for aquaculture, as salmon in groups always hold position against a current or swim voluntarily^24^.

In contrast, we caution against extrapolation beyond our model’s upper limit of 2.84 BL s^− 1^, representing 80–90% of group-specific critical swimming speed. At swimming speeds ≥ 80% of the critical swimming speed, salmonids begin transitioning from aerobic to anaerobic metabolism^64,65^, progressively recruiting higher proportions of fast-twitch glycolytic ‘white’ muscle over slow-twitch oxidative ‘red’ muscle^90–92^. Thus, beyond ~ 80% of the critical swimming speed, a declining proportion of swimming power is fuelled by oxygen^93,94^, explaining the plateau in MO_2_ observed near this limit^36,55^. This physiological plateau justifies our decision to exclude observations at 100% critical swimming speed from our refined dataset. Accordingly, extrapolation of our model beyond 80% of the critical swimming speed will almost certainly begin to overestimate MO_2_, so we recommend 80–90% as the functional ceiling for relative swimming speed extrapolation. In high-flow marine farming environments, oxygen is rapidly replenished by incoming water^16,17^, thereby minimising hypoxia risk and making exhaustion the primary welfare concern^33,71^.

#### Model predictions in hypoxic conditions

We expect our model to perform well under both normoxic and hypoxic conditions, with two caveats. First, predictions of MO_2_ should align with observations until reaching the limiting oxygen saturation (LOS). Empirical evidence shows that Atlantic salmon’s standard metabolic rate remains stable under hypoxia as long as sufficient oxygen is available to support the corresponding MO_2_^10,55,95^. Below these thresholds—described in detail by Remen et al.^10^ and Remen, Sievers, et al.^12^—MO_2_ becomes proportional to ambient DO, declining linearly with further reductions^9^. Thus, LOS increases linearly with MO_2_ across conditions^10^, and MO_2_ estimates at very low oxygen will almost certainly be overestimates, since MO_2_ cannot exceed the oxygen available, regardless of temperature or swimming speed. The second caveat concerns exercise intensity: while MO_2_ at low to moderate swimming speeds is unaffected by hypoxia, near critical swimming speeds hypoxia will suppress peak MO_2_^55^. We therefore advise caution when interpreting MO_2_ estimates at high relative swimming speeds under hypoxic conditions, as estimates may be inflated. These considerations are particularly important in sea cages, where moderate hypoxia is already common and oxygen can fluctuate rapidly from normoxia to hypoxia across both space and time^4,5,24,96–98^. Hypoxia is expected to become more frequent and severe in larger cages, at higher stocking densities, and under slack water flow^16,17,20,99,100^. Future farming sites may also encounter ambient oxygen saturation as low as 70–80%, leaving little scope for additional oxygen consumption before crossing into hypoxic conditions^19^.

### Limitations and future directions

While our model provides accurate estimates of MO_2_ across a broad and biologically relevant range of conditions, representing the full dynamic complexity of commercial aquaculture systems remains substantially more challenging. Oxygen consumption of farmed salmon is influenced by a wide array of interacting environmental and endogenous factors^23^, many of which differ markedly in mechanistic structure, temporal dynamics, and data availability. As such, it is neither practical nor conceptually appropriate to parameterise all such processes within a single core equation. Instead, the primary objective of this work was to establish a robust baseline formulation for MO_2_ of Atlantic salmon that can serve as a foundational component within digital simulation frameworks. This approach allows additional farm-relevant processes—such as feeding and digestion, production activities, or behavioural dynamics—to be incorporated as modular extensions of the simulation, rather than conflated within the core formulation. Our results further demonstrate that model departures from reality emerge during extrapolation and under specific farm-relevant conditions, underscoring the inherent limitations of attempting to represent such dynamic systems with a single predictive equation. While the present model provides reliable estimates under typical operating conditions and performs well within intermediate biological ranges, its accuracy can rapidly deteriorate near physiological limits and under extenuating conditions—precisely when accurate estimates are most urgent.

Integrating our revised MO_2_ model presented here into a digital twin framework^15^ would enable systematic incorporation of additional processes required to represent a broader spectrum of farming scenarios, including processes that cannot be adequately represented by standalone mathematical equations. Given the diversity of farm-relevant factors known to modulate MO_2_^23^, such a framework provides a flexible and scalable pathway for advancing from baseline physiological prediction toward fully integrated farm-scale simulations. For instance, the simulation developed and refined by Alver et al.^16,20,21^ already incorporates the dynamic spatial distribution of salmon driven by feeding behaviours and pellet distribution^101,102^, as well as avoidance of low oxygen levels^103^. Future iterations could integrate additional modulators of MO_2_ such as feeding and digestion^104,105^, photoperiod^106^, production activities like crowding and handling^107^, unsteady flows^108^, altered swimming behaviours across current speeds^109^, and the energetic toll of salmon lice and other prevailing pathogens^57,110^, among other factors^23^. Integrating these data into 3D digital simulations would enable more realistic representations of salmon MO_2_ in farming systems, revealing oxygen dynamics that could compromise welfare but are otherwise difficult or impossible to predict directly.

Our model can be further refined as additional group-level MO_2_ data becomes available through open-tank or swim tunnel respirometry, such as recent trials on 5–6 kg fish across different temperatures (Morin et al., unpublished observations). The main limitation of this study is the scarcity of data for fish above 1 kg. Thus, expanding group respirometry measurements to fish above 1 kg would be particularly valuable for improving our model’s predictive accuracy and industry relevance. Such improvements in data coverage will enable 3D oxygen dynamics simulations to be applied more effectively at full-scale, multi-cage commercial farms.

While our current model applies to seawater-adapted post-smolts, analogous models could be developed for earlier life stages—including fry, parr, and smolts—to provide a more comprehensive framework across the full production cycle^30^. We present our model using mass-specific metabolic rate (mg O_2_ kg^− 1^ h^− 1^) and relative swimming speed (BL s^− 1^), though it can be adapted to absolute oxygen consumption rate (mg O_2_ h^− 1^) and swimming speed (cm s^− 1^) when practical. Finally, because our model was developed using only AquaGen strain salmon, future work should test its validity across different genetic strains and geographical regions. Such refinements will strengthen this model’s generality and extend its utility across a broader range of aquaculture systems, research applications, and farm management scenarios.

## Conclusions

With our contemporary dataset and improved coefficient estimation approach, we recommend our refined fundamental model for oxygen consumption of Atlantic salmon (Eq. 3). Our model demonstrates strong predictive performance (adjusted R^2^ = 0.80) and more accurately reflects the metabolic demands of farmed Atlantic salmon:

3 $$\:{MO}_{2}=79.7{W}^{-0.14}1.0{4}^{T}1.{63}^{U}$$,

where MO_2_ is oxygen consumption rate (mg O_2_ kg^− 1^ h^− 1^), W is body weight (kg), T is water temperature (°C), and U is relative swimming speed (body lengths s^− 1^).

Our model represents an important and timely upgrade from prior empirical MO_2_ models, spanning an extensive range of metabolic rates (60–700 mg O_2_ kg^− 1^ h^− 1^), fish sizes (0.2–3.38 kg), water temperatures (3–18 °C), and relative swimming speeds (0.31–2.84 BL s^− 1^), thereby capturing the majority of conditions encountered in modern salmon farms. Using readily measurable parameters like body weight, water temperature, and relative swimming speed, our model offers broad utility across both fundamental research and aquaculture operations. For scientists, it enables refined predictions of metabolic demand across fish sizes, temperatures, and activity levels—supporting studies of bioenergetics, performance, and welfare. For engineers and farm operators, it provides a practical tool to estimate MO_2_ of large populations in diverse aquaculture systems. By relying on simple parameters, our model can be particularly valuable in forecasting hypoxia risk, establishing new aquaculture sites, and optimising environmental control in real time. Our model can also be useful in settings where direct measurements of oxygen consumption are impractical.

Our refined fundamental model delivers a more accurate and representative formula for predicting the oxygen demand of Atlantic salmon, establishing a stronger foundation for future oxygen dynamics simulations and enabling more precise, data-driven strategies for modern salmon aquaculture.

## Data Availability

The dataset and R script supporting the conclusions of this article are publicly available in the Zenodo repository: 10.5281/zenodo.19248487.

## References

[1] McIntosh, P. et al. Supersizing salmon farms in the coastal zone: A global analysis of changes in farm technology and location from 2005 to 2020. *Aquaculture* **553**, 738046 (2022).

[2] Moe Føre, H. et al. Technological innovations promoting sustainable salmon *(Salmo salar)* aquaculture in Norway. *Aquac Rep.* **24**, 101115 (2022).

[3] Farrell, A. P. Bulk oxygen uptake measured with over 60,000 kg of adult salmon during live-haul transportation at sea. *Aquaculture* **254**, 646–652 (2006).

[4] Johansson, D. et al. Effect of environmental factors on swimming depth preferences of Atlantic salmon (*Salmo salar* L.) and temporal and spatial variations in oxygen levels in sea cages at a fjord site. *Aquaculture* **254**, 594–605 (2006).

[5] Solstorm, D. et al. Dissolved oxygen variability in a commercial sea-cage exposes farmed Atlantic salmon to growth limiting conditions. *Aquaculture* **486**, 122–129 (2018).

[6] Tang, S., Brauner, C. J. & Farrell, A. P. Using bulk oxygen uptake to assess the welfare of adult Atlantic salmon, *Salmo salar*, during commercial live-haul transport. *Aquaculture* **286**, 318–323 (2009).

[7] Nilsson, J. et al. *Standardized Operational Welfare Monitoring for Salmon in Food Fish Farms*. (Norwegian Institute of Marine Research, 2022).

[8] Noble, C. et al. *Welfare Indicators for Farmed Atlantic Salmon: Tools for Assessing Fish Welfare*. (Norwegian Institute of Food, Fisheries and Aquaculture Research, 2018).

[9] Claireaux, G. & Chabot, D. Responses by fishes to environmental hypoxia: integration through Fry’s concept of aerobic metabolic scope. *J. Fish. Biol.* **88**, 232–251 (2016). 26768976 10.1111/jfb.12833

[10] Remen, M., Oppedal, F., Imsland, A. K., Olsen, R. E. & Torgersen, T. Hypoxia tolerance thresholds for post-smolt Atlantic salmon: Dependency of temperature and hypoxia acclimation. *Aquaculture* **416–417**, 41–47 (2013).

[11] Remen, M., Oppedal, F., Torgersen, T., Imsland, A. K. & Olsen, R. E. Effects of cyclic environmental hypoxia on physiology and feed intake of post-smolt Atlantic salmon: Initial responses and acclimation. *Aquaculture* **326–329**, 148–155 (2012).

[12] Remen, M., Sievers, M., Torgersen, T. & Oppedal, F. The oxygen threshold for maximal feed intake of Atlantic salmon post-smolts is highly temperature-dependent. *Aquaculture* **464**, 582–592 (2016).

[13] Neis, B. et al. Mass mortality events in marine salmon aquaculture and their influence on occupational health and safety hazards and risk of injury. *Aquaculture* **566**, 739225 (2023).

[14] Sajid, Z. et al. An aquaculture risk model to understand the causes and consequences of Atlantic Salmon mass mortality events: A review. *Rev. Aquac*. **16**, 1674–1695 (2024).

[15] Føre, M. et al. Digital Twins in intensive aquaculture — Challenges, opportunities and future prospects. *Comput. Electron. Agric.* **218**, 108676 (2024).

[16] Alver, M. O., Føre, M. & Alfredsen, J. A. Predicting oxygen levels in Atlantic salmon (*Salmo salar*) sea cages. *Aquaculture* **548**, 737720 (2022).

[17] Bergsson, H., Svendsen, M. B. S. & Steffensen, J. F. Model of Oxygen Conditions within Aquaculture Sea Cages. *Biology* **12**, 1408 (2023). 37998007 10.3390/biology12111408 PMC10669768

[18] Jónsdóttir, K. E. et al. Fish welfare based classification method of ocean current speeds at aquaculture sites. *Aquac Environ. Interact.* **11**, 249–261 (2019).

[19] Skagseth, Ø., Oppedal, F., Søiland, H. & Hvas, M. Measured oxygen levels in Norwegian waters and implications for future offshore Atlantic salmon aquaculture. *Sci. Rep.* **15**, 29416 (2025). 40790138 10.1038/s41598-025-12697-x PMC12339952

[20] Alver, M. O., Føre, M. & Alfredsen, J. A. Effect of cage size on oxygen levels in Atlantic salmon sea cages: A model study. *Aquaculture* **562**, 738831 (2023).

[21] Alver, M. O., Føre, M., Urke, H. A. & Alfredsen, J. A. Mathematical modelling of dissolved oxygen levels in a multi-cage salmon farm. *Aquaculture* **593**, 741291 (2024).

[22] Russell, W. M. S., Burch, R. L. & Hume, C. *W. The Principles of Humane Experimental Technique.* (Methuen, 1959).

[23] Berntsson, E. V. C. et al. Managing the Dissolved Oxygen Balance of Open Atlantic Salmon Sea Cages: A Narrative Review. *Rev. Aquac*. **17**, e12992 (2025).

[24] Oppedal, F., Dempster, T. & Stien, L. H. Environmental drivers of Atlantic salmon behaviour in sea-cages: A review. *Aquaculture* **311**, 1–18 (2011).

[25] Clarke, A. & Johnston, N. M. Scaling of metabolic rate with body mass and temperature in teleost fish. *J. Anim. Ecol.* **68**, 893–905 (1999).

[26] Forsberg, O. I. Modelling oxygen consumption rates of post-smolt Atlantic salmon in commercial-scale, land-based farms. *Aquac Int.* **2**, 180–196 (1994).

[27] Kraskura, K., Patterson, D. A. & Eliason, E. J. A review of adult salmon maximum swim performance. *Can. J. Fish. Aquat. Sci.* **81**, 1174–1216 (2024).

[28] Thorarensen, H. & Farrell, A. P. The biological requirements for post-smolt Atlantic salmon in closed-containment systems. *Aquaculture* **312**, 1–14 (2011).

[29] Grøttum, J. A. & Sigholt, T. A model for oxygen consumption of Atlantic salmon (*Salmo salar*) based on measurements of individual fish in a tunnel respirometer. *Aquac Eng.* **17**, 241–251 (1998).

[30] Macnaughton, C. J., Deslauriers, D., Ipsen, E. L., Corey, E. & Enders, E. C. Using meta-analysis to derive a respiration model for Atlantic salmon (*Salmo salar*) to assess bioenergetics requirements of juveniles in two Canadian rivers. *Can. J. Fish. Aquat. Sci.* **76**, 2225–2234 (2019).

[31] Chabot, D., Steffensen, J. F. & Farrell, A. P. The determination of standard metabolic rate in fishes. *J. Fish. Biol.* **88**, 81–121 (2016). 26768973 10.1111/jfb.12845

[32] Norin, T. & Clark, T. D. Measurement and relevance of maximum metabolic rate in fishes. *J. Fish. Biol.* **88**, 122–151 (2016). 26586591 10.1111/jfb.12796

[33] Hvas, M. & Oppedal, F. Sustained swimming capacity of Atlantic salmon. *Aquac Environ. Interact.* **9**, 361–369 (2017).

[34] Gjedrem, T., Gjøen, H. M. & Gjerde, B. Genetic origin of Norwegian farmed Atlantic salmon. *Aquaculture* **98**, 41–50 (1991).

[35] Remen, M. et al. Critical swimming speed in groups of Atlantic salmon *Salmo salar*. *Aquac Environ. Interact* **8**, 659–664 (2016).

[36] Hvas, M., Folkedal, O., Imsland, A. & Oppedal, F. The effect of thermal acclimation on aerobic scope and critical swimming speed in Atlantic salmon, *Salmo salar*. *J. Exp. Biol.* **220**, 2757–2764 (2017). 28507190 10.1242/jeb.154021

[37] Hvas, M. & Oppedal, F. Influence of experimental set-up and methodology for measurements of metabolic rates and critical swimming speed in Atlantic salmon *Salmo salar*. *J. Fish. Biol.* **95**, 893–902 (2019). 31265133 10.1111/jfb.14087

[38] Morin, A. et al. Tank- and cage-farmed Atlantic salmon display similar swimming performance and appetite recovery despite differences in gill and heart morphology. *Aquaculture* **609**, 742826 (2025).

[39] Gjedrem, T. Genetic improvement for the development of efficient global aquaculture: A personal opinion review. *Aquaculture* **344–349**, 12–22 (2012).

[40] Janssen, K., Chavanne, H., Berentsen, P. & Komen, H. Impact of selective breeding on European aquaculture. *Aquaculture* **472**, 8–16 (2017).

[41] Næve, I., Korsvoll, S. A., Santi, N., Medina, M. & Aunsmo, A. The power of genetics: Past and future contribution of balanced genetic selection to sustainable growth and productivity of the Norwegian Atlantic salmon (*Salmo salar*) industry. *Aquaculture* **553**, 738061 (2022).

[42] Oppedal, F., Vågseth, T., Dempster, T., Juell, J. E. & Johansson, D. Fluctuating sea-cage environments modify the effects of stocking densities on production and welfare parameters of Atlantic salmon (*Salmo salar* L). *Aquaculture* **315**, 361–368 (2011).

[43] Brown, J. H., Gillooly, J. F., Allen, A. P., Savage, V. M. & West, G. B. Toward a Metabolic Theory of Ecology. *Ecology* **85**, 1771–1789 (2004).

[44] Calder, W. A. *Size Function, and Life History.* (Harvard University Press, 1984).

[45] Kleiber, M. *The Fire of Life: An Introduction to Animal Energetics.* (John Wiley & Sons, Inc., 1961).

[46] Peters, R. H. *The Ecological Implications of Body Size.* (Cambridge University Press, 1983).

[47] Schmidt-Nielsen, K. *Scaling: Why Is Animal Size so Important?.* (Cambridge University Press, 1984).

[48] Clarke, A. & Fraser, K. P. P. Why Does Metabolism Scale with Temperature?. *Funct. Ecol.* **18**, 243–251 (2004).

[49] Arrhenius, S. On the reaction rate of the inversion of non-refined sugar upon souring. *J. Phys. Chem.* **4**, 226–248 (1889).

[50] Arrhenius, S. *Quantitative Laws in Biological Chemistry.* (G. Bell and Sons, Ltd., 1916).

[51] van ’t Hoff, J. H. *Studies in Chemical Dynamics*. (F. Müller and Co, Amsterdam, 1896).

[52] Brett, J. R. The respiratory metabolism and swimming performance of young sockeye salmon. *J. Fish. Res. Board. Can.* **21**, 1183–1226 (1964).

[53] Videler, J. J. *Fish Swimming.* (Springer Netherlands, 1993).

[54] Webb, P. W. *Hydrodynamics and Energetics of Fish Propulsion. *(Fisheries Research Board of Canada, 1975).

[55] Oldham, T., Nowak, B., Hvas, M. & Oppedal, F. Metabolic and functional impacts of hypoxia vary with size in Atlantic salmon. *Comp. Biochem. Physiol. Mol. Integr. Physiol.* **231**, 30–38 (2019). 10.1016/j.cbpa.2019.01.012 30690152

[56] Hvas, M. Swimming energetics of Atlantic salmon in relation to extended fasting at different temperatures. *Conserv. Physiol.* **10**, coac037 (2022). 35733620 10.1093/conphys/coac037 PMC9208137

[57] Hvas, M., Karlsbakk, E., Mæhle, S., Wright, D. W. & Oppedal, F. The gill parasite *Paramoeba perurans* compromises aerobic scope, swimming capacity and ion balance in Atlantic salmon. *Conserv Physiol* **5**, cox066 (2017). 10.1093/conphys/cox066 PMC5710617 29218225

[58] Hvas, M., Nilsen, T. O. & Oppedal, F. Oxygen Uptake and Osmotic Balance of Atlantic Salmon in Relation to Exercise and Salinity Acclimation. *Front Mar. Sci* **5**, 368 (2018).

[59] Hvas, M., Folkedal, O. & Oppedal, F. What is the limit of sustained swimming in Atlantic salmon post smolts?. *Aquac Environ. Interact.* **13**, 189–198 (2021).

[60] R Core Team. *R: The R Project for Statistical Computing.* (R Foundation for Statistical Computing, 2025).

[61] Pinheiro, J., Bates, D. & Core Team. R. *nlme: Linear and Nonlinear Mixed Effects Models*. (2025).

[62] Glazier, D. S. Beyond the ‘3/4-power law’: variation in the intra-and interspecific scaling of metabolic rate in animals. *Biol. Rev.* **80**, 611–662 (2005). 16221332 10.1017/S1464793105006834

[63] Glazier, D. S. Activity affects intraspecific body-size scaling of metabolic rate in ectothermic animals. *J. Comp. Physiol. B*. **179**, 821–828 (2009). 19387653 10.1007/s00360-009-0363-3

[64] Burgetz, I. J., Rojas-Vargas, A., Hinch, S. G. & Randall, D. J. Initial Recruitment of Anaerobic Metabolism During Sub-Maximal Swimming in Rainbow Trout (*Oncorhynchus mykiss*). *J. Exp. Biol.* **201**, 2711–2721 (1998). 9732326 10.1242/jeb.201.19.2711

[65] Geist, D. R. et al. Relationships between metabolic rate, muscle electromyograms and swim performance of adult chinook salmon. *J. Fish. Biol.* **63**, 970–989 (2003).

[66] Mowi. *Salmon Farming Industry Handbook*. (2024).

[67] Barrett, L. T., Oldham, T., Kristiansen, T. S., Oppedal, F. & Stien, L. H. Declining size-at-harvest in Norwegian salmon aquaculture: Lice, disease, and the role of stunboats. *Aquaculture* **559**, 738440 (2022).

[68] Falconer, L. et al. Marine aquaculture sites have huge potential as data providers for climate change assessments. *Aquaculture* **595**, 741519 (2025).

[69] Korus, J., Filgueira, R. & Grant, J. Influence of temperature on the behaviour and physiology of Atlantic salmon (*Salmo salar*) on a commercial farm. *Aquaculture* **589**, 740978 (2024).

[70] Hansen, T. J., Fjelldal, P. G., Folkedal, O., Vågseth, T. & Oppedal, F. Effects of light source and intensity on sexual maturation, growth and swimming behaviour of Atlantic salmon in sea cages. *Aquac Environ. Interact.* **9**, 193–204 (2017).

[71] Hvas, M., Folkedal, O. & Oppedal, F. Fish welfare in offshore salmon aquaculture. *Rev. Aquac*. **13**, 836–852 (2021).

[72] Juell, J. E. The behaviour of Atlantic salmon in relation to efficient cage-rearing. *Rev. Fish. Biol. Fish.* **5**, 320–335 (1995).

[73] Oppedal, F., Bui, S., Stien, L. H., Overton, K. & Dempster, T. Snorkel technology to reduce sea lice infestations: efficacy depends on salinity at the farm site, but snorkels have minimal effects on salmon production and welfare. *Aquac Environ. Interact.* **11**, 445–457 (2019).

[74] Sutterlin, A. M., Jokola, K. J. & Holte, B. Swimming Behavior of Salmonid Fish in Ocean Pens. *J. Fish. Res. Board. Can.* **36**, 948–954 (1979).

[75] Warren-Myers, F. et al. Full production cycle, commercial scale culture of salmon in submerged sea-cages with air domes reduces lice infestation, but creates production and welfare challenges. *Aquaculture* **548**, 737570 (2022).

[76] Warren-Myers, F. et al. Efficiency of salmon production in submerged cages with air domes matches standard surface cages when environments are similar. *Aquaculture* **586**, 740751 (2024).

[77] Ernest, S. K. M. et al. Thermodynamic and metabolic effects on the scaling of production and population energy use. *Ecol. Lett.* **6**, 990–995 (2003).

[78] Gillooly, J. F., Brown, J. H., West, G. B., Savage, V. M. & Charnov, E. L. Effects of size and temperature on metabolic rate. *Science* **293**, 2248–2251 (2001). 11567137 10.1126/science.1061967

[79] West, G. B., Brown, J. H. & Enquist, B. J. A General Model for the Origin of Allometric Scaling Laws in Biology. *Science* **276**, 122–126 (1997). 9082983 10.1126/science.276.5309.122

[80] West, G. B. & Brown, J. H. The origin of allometric scaling laws in biology from genomes to ecosystems: towards a quantitative unifying theory of biological structure and organization. *J. Exp. Biol.* **208**, 1575–1592 (2005). 15855389 10.1242/jeb.01589

[81] Buller, F. *The Domesday Book of Giant Salmon* (Little, Brown Book Group Limited, 2007).

[82] Gerber, L., MacSween, C. E., Staples, J. F. & Gamperl, A. K. Cold-induced metabolic depression in cunner (*Tautogolabrus adspersus*): A multifaceted cellular event. *PLOS ONE*. **17**, e0271086 (2022). 35917356 10.1371/journal.pone.0271086 PMC9345476

[83] Porter, E. S. & Gamperl, A. K. Cardiorespiratory physiology and swimming capacity of Atlantic salmon (*Salmo salar*) at cold temperatures. *J. Exp. Biol.* **226**, jeb245990 (2023). 37661722 10.1242/jeb.245990 PMC10499030

[84] Porter, E. S., Clow, K. A., Sandrelli, R. M. & Gamperl, A. K. Acute and chronic cold exposure differentially affect cardiac control, but not cardiorespiratory function, in resting Atlantic salmon (*Salmo salar*). *Curr. Res. Physiol.* **5**, 158–170 (2022). 35359619 10.1016/j.crphys.2022.03.002 PMC8960890

[85] Fletcher, G. L., Kao, M. H. & Dempson, J. B. Lethal freezing temperatures of Arctic char and other salmonids in the presence of ice. *Aquaculture* **71**, 369–378 (1988).

[86] Andrew, S., Currie, S. & Morash, A. J. The effects of warm thermal variability on metabolism and swimming performance in wild Atlantic salmon (*Salmo salar*). *J. Fish. Biol.* **106**, 893–907 (2025). 39581221 10.1111/jfb.15996 PMC11949746

[87] Hvas, M., Morin, A., Johansen, I. B. & Vågseth, T. Acute stress-induced mortality in big Atlantic salmon at high temperatures is associated with insufficient oxygen uptake capacity. *J. Therm. Biol.* **132**, 104231 (2025). 40829375 10.1016/j.jtherbio.2025.104231

[88] Gamperl, A. K. et al. The impacts of increasing temperature and moderate hypoxia on the production characteristics, cardiac morphology and haematology of Atlantic Salmon (*Salmo salar*). *Aquaculture* **519**, 734874 (2020).

[89] Beamish, F. W. H. 2 - Swimming Capacity in *Fish Physiology* (eds. Hoar, W. S. & Randall, D. J.) 101–187 (Academic Press, 1978).

[90] Egginton, S. & Sidell, B. D. Thermal acclimation induces adaptive changes in subcellular structure of fish skeletal muscle. *Am. J. Physiol. -Regul Integr. Comp. Physiol.* **256**, R1–R9 (1989). 10.1152/ajpregu.1989.256.1.R1 2912202

[91] McKenzie, D. J. Swimming and other activities | Energetics of Fish Swimming in *Encyclopedia of Fish Physiology* (ed. Farrell, A. P.) 1636–1644 (Academic Press, 2011).

[92] Sänger, A. M. & Stoiber, W. 7 - Muscle Fiber Diversity and Plasticity in* Fish Physiology *(ed. Johnston, I. A.) 187–250 (Academic Press, 2001).

[93] Brett, J. R. The Relation of Size to Rate of Oxygen Consumption and Sustained Swimming Speed of Sockeye Salmon (*Oncorhynchus nerka*). *J. Fish. Res. Board. Can.* **22**, 1491–1501 (1965).

[94] Brett, J. R. Energy Expenditure of Sockeye Salmon, *Oncorhynchus nerka*, During Sustained Performance. *J. Fish. Res. Board. Can.* **30**, 1799–1809 (1973).

[95] Hvas, M. & Oppedal, F. Physiological responses of farmed Atlantic salmon and two cohabitant species of cleaner fish to progressive hypoxia. *Aquaculture* **512**, 734353 (2019).

[96] Burt, K. et al. Environmental conditions and occurrence of hypoxia within production cages of Atlantic salmon on the south coast of Newfoundland. *Aquac Res.* **43**, 607–620 (2012).

[97] Jeong, J. et al. Longitudinal dissolved oxygen patterns in Atlantic salmon aquaculture sites in British Columbia, Canada. *Front Mar. Sci* **10**, 1289375 (2024).

[98] Johansson, D., Juell, J. E., Oppedal, F., Stiansen, J. E. & Ruohonen, K. The influence of the pycnocline and cage resistance on current flow, oxygen flux and swimming behaviour of Atlantic salmon (*Salmo salar* L.) in production cages. *Aquaculture* **265**, 271–287 (2007).

[99] Oldham, T., Oppedal, F. & Dempster, T. Cage size affects dissolved oxygen distribution in salmon aquaculture. *Aquac Environ. Interact.* **10**, 149–156 (2018).

[100] Wen, X., Ong, M. C., Yin, G., Ludvigsen, T. & Oppedal, F. Influence of Ocean Currents and Stocking Density on the Dissolved Oxygen Distribution Inside Gravity-Type Fish Cages. *J Offshore Mech. Arct. Eng* **147**, 061301 (2025).

[101] Alver, M. O., Alfredsen, J. A. & Sigholt, T. Dynamic modelling of pellet distribution in Atlantic salmon (*Salmo salar* L.) cages. *Aquac Eng.* **31**, 51–72 (2004).

[102] Alver, M. O. et al. Modelling of surface and 3D pellet distribution in Atlantic salmon (*Salmo salar* L.) cages. *Aquac Eng.* **72–73**, 20–29 (2016).

[103] Stehfest, K. M., Carter, C. G., McAllister, J. D., Ross, J. D. & Semmens, J. M. Response of Atlantic salmon *Salmo salar* to temperature and dissolved oxygen extremes established using animal-borne environmental sensors. *Sci. Rep.* **7**, 4545 (2017). 28674437 10.1038/s41598-017-04806-2 PMC5495760

[104] Brett, J. R. 10 - Environmental Factors and Growth in *Fish Physiology* (eds. Hoar, W. S., Randall, D. J. & Brett, J. R.) 599–675 (Academic Press, 1979).

[105] Forsberg, O. I. The impact of varying feeding regimes on oxygen consumption and excretion of carbon dioxide and nitrogen in post-smolt Atlantic salmon *Salmo salar* L. *Aquac Res.* **28**, 29–41 (1997).

[106] Folkedal, O., Torgersen, T., Nilsson, J. & Oppedal, F. Habituation rate and capacity of Atlantic salmon (*Salmo salar*) parr to sudden transitions from darkness to light. *Aquaculture* **307**, 170–172 (2010).

[107] Forsberg, O. I. Oxygen consumption of post-smolt Atlantic salmon during crowding and handling stress. *Aquac Int.* **3**, 55–59 (1995).

[108] Agbeti, W. E. K. et al. Swimming at Increasing Speeds in Steady and Unsteady Flows of Atlantic Salmon *Salmo salar*: Oxygen Consumption, Locomotory Behaviour and Overall Dynamic Body Acceleration. *Biology* **13**, 393 (2024). 38927273 10.3390/biology13060393 PMC11200746

[109] Johansson, D. et al. The Interaction between Water Currents and Salmon Swimming Behaviour in Sea Cages. *PLOS ONE*. **9**, e97635 (2014). 24830443 10.1371/journal.pone.0097635 PMC4022588

[110] Hvas, M. & Bui, S. Energetic costs of ectoparasite infection in Atlantic salmon. *J. Exp. Biol.* **225**, jeb243300 (2022). 34931653 10.1242/jeb.243300

